# Structural approaches for prevention of sexually transmitted HIV in general populations: definitions and an operational approach

**DOI:** 10.7448/IAS.17.1.19052

**Published:** 2014-09-08

**Authors:** Justin O Parkhurst

**Affiliations:** Department of Global Health and Development, London School of Hygiene and Tropical Medicine, London, United Kingdom

**Keywords:** structural drivers, social drivers, structural approaches, HIV prevention, Implementation

## Abstract

**Introduction:**

Although biomedical HIV prevention efforts have seen a number of recent promising developments, behavioural interventions have often been described as failing. However, clear lessons have been identified from past efforts, including the need to address influential social, economic and legal structures; to tailor efforts to local contexts; and to address multiple influencing factors in combination. Despite these insights, there remains a pervasive strategy to try to achieve sexual behaviour change through single, decontextualized, interventions or sets of activities. With current calls for structural approaches to HIV as part of combination HIV prevention, though, there is a unique opportunity to define a structural approach to HIV prevention as one which moves beyond these past limitations and better incorporates our knowledge of the social world and the lessons from past efforts.

**Discussion:**

A range of interlinked concepts require delineation and definition within the broad concept of a structural approach to HIV. This includes distinguishing between “structural factors,” which can be seen as any number of elements (other than knowledge) which influence risk and vulnerability, and “structural drivers,” which should be reserved for situations where an empirically established relationship to a target group is known. Operationalizing structural approaches similarly can take different paths, either working to alter structural drivers or alternatively working to build individual and community resilience to infection. A “structural diagnostic approach” is further defined as the process one undertakes to develop structural intervention strategies tailored to target groups.

**Conclusions:**

For three decades, the HIV prevention community has struggled to reduce the spread of HIV through sexual risk behaviours with limited success, but equally with limited engagement with the lessons that have been learned about the social realities shaping patterns of sexual practices. Future HIV prevention efforts must address the multiple factors influencing risk and vulnerability, and they must do so in ways tailored to particular settings. Clarity on the concepts, terminology and approaches that can allow structural HIV prevention efforts to achieve this is therefore essential to improve the (social) science of HIV prevention.

## Introduction – improving the science of HIV prevention

The HIV prevention field has seen remarkable progress in recent years on the biomedical front, with the promise of drug- or surgery-based prevention strategies such as male circumcision, pre-exposure prophylaxis and early initiation of widespread antiretroviral therapy (“test and treat”) [1–5]. Unfortunately, there have been fewer clear examples of behavioural interventions which have been shown to sustainably bring about reductions in HIV incidence [6–8]. There have been arguments that the behavioural/biomedical divide itself may undermine prevention efforts, as the majority of interventions classified as “biomedical” require human behaviours and/or structural shifts to change to ensure their success (e.g., pre-exposure prophylaxis requires regular taking of a pill each day, analogous to the way condoms must be worn regularly to ensure the full benefit of their protective effect) [9, 10]. Indeed, Kippax and Stephenson [10] lament how the lack of successes measured to bring about sustained HIV incidence reduction through single strategies has often led to claims that HIV prevention has “failed” [10], even though there are obvious cases of population groups who have, in fact, altered their risk practices to respond to, and reduce HIV infections (pointing to Australian, Ugandan and Zimbabwean examples) [10].

Therefore, although many prevention interventions may have proved wanting, after 30 years of the fight against the HIV/AIDS epidemic, a number of clear lessons can be learned.

First, there is understanding that the patterning of human sexual practices are deeply embedded in, and shaped by, underlying social, economic and legal-political structures [6, 7, 9, 11–13]. Reducing HIV risk, therefore, will typically require changes in broader structural elements (be they economic opportunities, social norms and gender roles, legal freedoms or a combinations of these factors), not just information provision alone [12, 14–18]. Second, it is recognized that much HIV prevention activity has occurred without sufficient conceptualization of why or how a particular approach should actually bring about a sustained change in behaviour in a given setting [19–21], with current calls by the Joint United Nations Programme on HIV/AIDS (UNAIDS) and others to tailor HIV responses to the factors shaping risk and vulnerability in specific contexts [13, 18, 21–23]. Third, human behaviours are not determined by single causal factors, but rather by multiple elements in combination, which influence patterning of risk behaviour. (See Heise and Watts [24] for a discussion of how multiple risk-increasing practices may often cluster together, and therefore may need to be addressed in combination – for example, how violent behaviour towards female partners is commonly linked with excessive alcohol consumption and frequenting of sex workers, and Aral [8] for a discussion of the complex behaviour-systems in which individual behaviours are embedded.)

Some recent works have found that single-component or “one-off” interventions can indeed reduce behavioural risks for individuals, at least in the short term [25] (with the Zomba cash-transfer trial providing some of the most impressive results to date [26]), but single behavioural interventions cannot alter social structures that provide the environment in which patterns of risk practices are developed (sometimes over generations). Long-term, sustained alteration of these patterns instead requires a more comprehensive approach to structural change [12, 14, 18, 20, 27–34]. Indeed, Wellings *et al*. [34], from a review of sexual behaviour data from 59 countries, conclude: “Evidence from behavioural interventions shows that no general approach to sexual-health promotion will work everywhere and no single-component intervention is likely to work anywhere” (p. 1724).

These insights have supported current calls for “combination HIV prevention” approaches, defined by UNAIDS as “simultaneous use of complementary behavioural, biomedical and structural prevention strategies” [21, p. 5]. Yet these insights are in no way new. The need for more than information (including HIV knowledge) to affect HIV risk behaviour has been known since at least the late 1980s [16, 32, 35, 36]. The importance of tailored HIV prevention strategies was also clearly stated two decades ago in reviews of both African [29] and American [37] HIV prevention experiences. Similarly, the importance of addressing broader structures was the subject an entire supplement of the journal *AIDS* in 2000 [38], part of a *Lancet* series released in June 2012 [12], and was a thematic area of the aids 2031 programme [39].

Despite countless journal articles making the above points about the need to look beyond information provision and address wider structures, national AIDS responses cling determinedly to information, education and communication programmes, while the biomedical research community has maintained a hope that a single (decontextualized), predefined intervention targeting behaviour can be tested in an experimental trial that might lead to significant and sustained changes in risk practices [18, 40, 41]. Such thinking flies in the face of all that has been learned about factors influencing patterns of sexual behaviours in populations [18, 28, 42]. The lessons of the past have pointed to three key objectives that future behaviour-change-based prevention efforts must therefore work to achieve:

1. To address broader structures shaping behavioural risk and vulnerability;
2. To tailor responses to the factors influencing risk and vulnerability understood to affect the target population;
3. To ensure multiple factors can be addressed when needed.

When we have seen success stories in particular population groups – such as sex workers in Kolkata [43, 44], or gay men in the west [27] – these have typically not been achieved through predefined “interventions” but rather by responding to local needs in a tailored, bottom-up direction through approaches that actively engage with the target populations’ reasons for their behaviours [27]. Sociologically informed works note the importance of individual and community agency in affecting how populations react to any changes in their environment, emphasizing the fact that individuals undertake behaviours and construct practices because they have their own reasons for doing so (and not because they are unconsciously responding to external stimuli) [27, 45, 46]. As such Adam [27] asks “why can there not be prevention knowledge that starts from the grounded experience of people who deal most directly with HIV risk …?” (p. 5). One reason why this question is rarely asked is that it is tempting, even intuitive from a natural science perspective, to look at past successes and attempt to copy the activities conducted. But activities applied from other settings do not achieve the above three objectives on their own. Instead, given the nature of social change, what is more critical is to copy the approach taken.

What the science of HIV prevention has yet to develop are generalizable strategies to provide what target groups need in tailored ways, ways which respond to the specific set of multiple structural factors influencing the groups’ risk and vulnerability. We have yet to see, for example, randomized trials or operational research evaluating processes (e.g., ways of engaging with populations or ways of identifying local needs) rather than predefined interventions. The failure of three decades of AIDS prevention efforts to develop top-down interventions which can achieve significant and sustained changes in behaviour, and the failure, seemingly, to incorporate the lessons that repeated reviews of behaviour change and examinations of real-life successes have shown, should be a clear wake-up call for the need to approach HIV behaviour change differently. Achieving such a shift away from top-down de-contextualized approaches to HIV prevention would be nothing short of revolutionary, but defining a structural approach to HIV as one which incorporates the three objectives above would be an important first step.

## Discussion

### Definitions and concepts

Often, the term “structural” is taken to mean in effect, anything more than the individual. In this conceptualization, everything has structural influences – from human behaviour, to health systems functioning, to the determinants of biomedical research funding priorities. Such a broad conceptualization, however, inherently reduces the operational usefulness of the term. The consideration of structural factors and the recognition of the locally specific and dynamic ways in which they influence behaviour are strongly rooted in sociological theory about how human actions and choices are related to broader influences. Understanding this complex linkage has never been easy – it has been termed one of the “central problems in social theory” [47] and has been the subject of theorizing for more than a hundred years (as seen in the development of such bodies of theory as structuralism, functionalism, structural-functionalism, structuration and post-structuralism) [48] – but drawing on insights from social theory provides a conceptual starting point from which to consider critical elements and processes with which a structural approach to HIV prevention might engage.

Within this broad, social science-informed approach, there are two basic ways in which authors discuss structural HIV prevention. The first body of work conceptualizes structural factors as those which fundamentally shape or influence patterns of risk behaviour – the drivers [12, 14, 21, 39, 41, 49] – whereas the second group conceptualizes structures as environmental factors which facilitate or hinder (i.e., mediate) how people can specifically avoid HIV within a given context [6, 38, 49, 50]. Conceptualizing structural factors in these two ways (as risk drivers or as environmental barriers/facilitators) provides an important first step to guide locally tailored intervention strategies.

### Drivers and mediators

Conceptualizing structural factors as either drivers of behaviour or mediators of risk is a first step in moving beyond the oversimplified HIV prevention strategies of the past – to ensure broader structures are considered, responses are tailored, and multiple interacting factors are considered. The language of “drivers” particularly appeals within the public health community, whose members are accustomed to looking for causal determinants of illness. A risk with this language is that it can lead to an oversimplified view of causality. Abundant research has shown that linear causality from single determinants almost never exists for patterns of behaviour, and the direction or magnitude of effect can vary over place and time [41]. The language of drivers also risks downplaying the importance of human agency, and the fact that within any structural environment, communities of individuals will construct their own sets of practices. To address the risks of oversimplification, it is critical to use the language of structural drivers only in context-specific ways; preferably with empirical evidence or information identifying how and why groups have constructed or chosen practices in a specific structural setting. Structural “factors” can be seen as a broader concept, encompassing the multitude of potential elements which might shape the patterning of risk and vulnerability for different populations, whereas structural drivers would encompass an identified set of factors empirically identified to be important in influencing the risk practices of a given target group. Emphasizing the need to empirically validate a driver before attempts to intervene can help to ensure local tailoring in HIV responses.

The alternative conceptualization has been to approach structures as environmental factors that affect which safe behaviours can be chosen. In this way, the emphasis is less on the factors that influence sexual networks or relationships, and more on HIV prevention considerations and the capability of individuals to act with HIV prevention in mind. Sumartojo *et al*. [38], for example, defines “HIV related structural factors … as barriers to, or facilitators of, an individual's HIV prevention behaviours” (p. 3). The AIDS 2031 Social Drivers Working Group has alternatively defined a structural approach as one which builds “AIDS resilience” – achieved when individuals possess the ability to resist HIV, and their environment is conducive to HIV prevention. As with the risk driver approach, an environmental conceptualization again requires tailoring, as there will not be a single environment that supports HIV prevention, and the elements which facilitate or hinder safe behaviours need to be addressed locally.

### Pathways and levels

**Figure 1:**
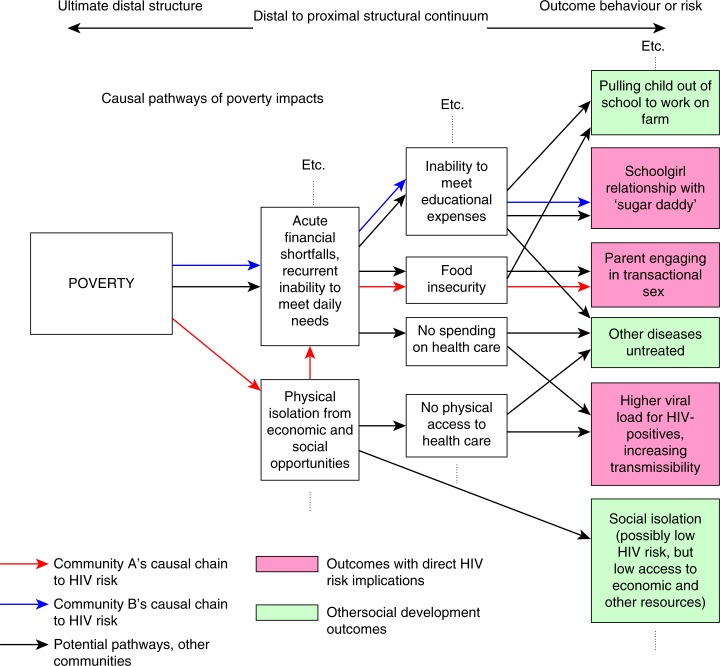
Example of causal pathways from poverty to HIV risk.

The understanding of structural factors as risk drivers has also led to consideration of the causal pathways through which structural factors may manifest in HIV transmission events, and the levels at which organizations might look to respond. A hypothetical example, in part adapted from Gupta *et al*. [12], is presented below (Figure 1) to illustrate the causal pathways through which poverty might manifest in risk differently (or not at all) in different settings.

Figure 1 maps out causal pathways as moving from upstream, “distal” influences to more downstream, “proximal” influences [51] An organization concerned with addressing poverty to reduce HIV risk could consider multiple points of intervention. But in doing so, it must take a tailored approach which empirically establishes how poverty actually manifests in HIV risk in the target population [52]. This can be seen as the “mechanism of effect” through which poverty is linked to HIV risk in a given setting, and must be explicitly considered in structural HIV prevention approaches. It is also worth note that causal pathways of this sort will also be dynamic and may change over time. We have seen examples where diseases first associated with wealthy lifestyles have shifted to increasing burdens in the poor in particular locales (e.g., obesity, smoking related illness, or, indeed, HIV/AIDS [53–55]). As such, programme planners will want to reflect on the empirical data used to establish hypothesized causal pathways, and how likely it is that elements within the pathways may have changed (in the example in Figure 1, this could mean considering if there have been recent shifts in economic activities locally, if new government programmes have changed access to education or health care, if social practices in response to poverty have evolved recently, etc.).

In addition to the causal pathway, a related concept is to consider the various levels at which structures exist, to help identify where an organization might look to intervene. Macro factors, for example, can be seen as those that affect entire nations or regions (such as national economic policies or legal frameworks). Meso-level factors, alternatively, are those that shape group- and community-level elements (such as gender and behavioural norms, or religious beliefs). Finally, micro-level structures are those that influence individuals or family units (such as economic vulnerability or lack of education) [39, 56, 57]. Frameworks which consider levels of influence are often described using so-called “ecological” models that present individuals sitting in nested layers of influence (illustrated as concentric circles [58, 59] or as resembling the layers of an onion [13]).

The importance of proximity and level of influence have particular relevance to implementation of structural strategies. Proximal interventions typically have more direct cause-effect relationships and may see more immediate results. They may be limited, however, in the number of risk-shaping factors that they can target, and they may not be sufficient to achieve significant or sustained changes in patterns of risk behaviour on their own. Upstream, distal changes may lead to long-term shifts in patterns of behaviour, or may affect multiple factors, but tend to do so in very indirect ways, and may require long periods of time to realize their effects [39, 41] These realities may prove particularly challenging to implementing organizations. A recent article by Hunsmann [60], for example, illustrates how the existing HIV response structures of many donor and government organizations are not conducive to actually engaging with the more distal, less immediate influences shaping HIV risk, nor are they designed to be able to address multiple causal elements [60]. These insights help explain why much of what is needed to move HIV prevention forward – addressing broader structures, using tailored interventions and addressing multiple causal elements – has been known for decades, yet has not been taken up. The article shows that, in the case of Tanzania at least, the problem may lie as much, or even more so, in the institutional structures of the agencies responding, rather than in any lack of evidence or knowledge of what is needed.

### Structural “interventions” and “approaches”

Indeed, throughout the history of HIV prevention, donors, governments and implementing agencies alike have typically tried to identify predetermined “interventions” that include guidelines or clear steps for implementation. With recent calls to consider structural factors, there has equally been concern to identify a “set” of structural interventions that might “work” for HIV prevention. As discussed above, however, this search for decontextualized interventions has seriously limited HIV prevention in the past by failing to respond to broader structures in tailored ways, or by failing to address the multifaceted nature of risk and vulnerability.

Rather than a predefined, off-the-shelf application of interventions, what is needed is an approach to ensure that the best possible package of interventions is selected for the local target population. The intervention strategy, and choice of actual activities, will need to be the result of a process that identifies relevant structural drivers or influences, considers the ways a recipient community may respond to interventions and tailors the response to the multiple needs of the target population in a way that is feasible for the implementing agency (typically based on the level at which the agency is capable of intervening). A structural approach to HIV can therefore be defined as the process undertaken to decide upon an appropriate set of structural HIV prevention interventions: a *process* because it is impossible to define in advance what activities to undertake; *appropriate* because HIV prevention must be tailored to local realities; and a *set* of activities because risk is typically shaped by multiple factors. In this way, a structural approach can be conceptualized as a decision tree, where a series of questions is answered, or a series of steps is taken, to ultimately arrive at an intervention and evaluation strategy. Box 1 attempts to provide a summary of definitions of terms used that may help in the operationalization of such an approach.

### Operationalizing a “structural approach”

**Figure 2:**
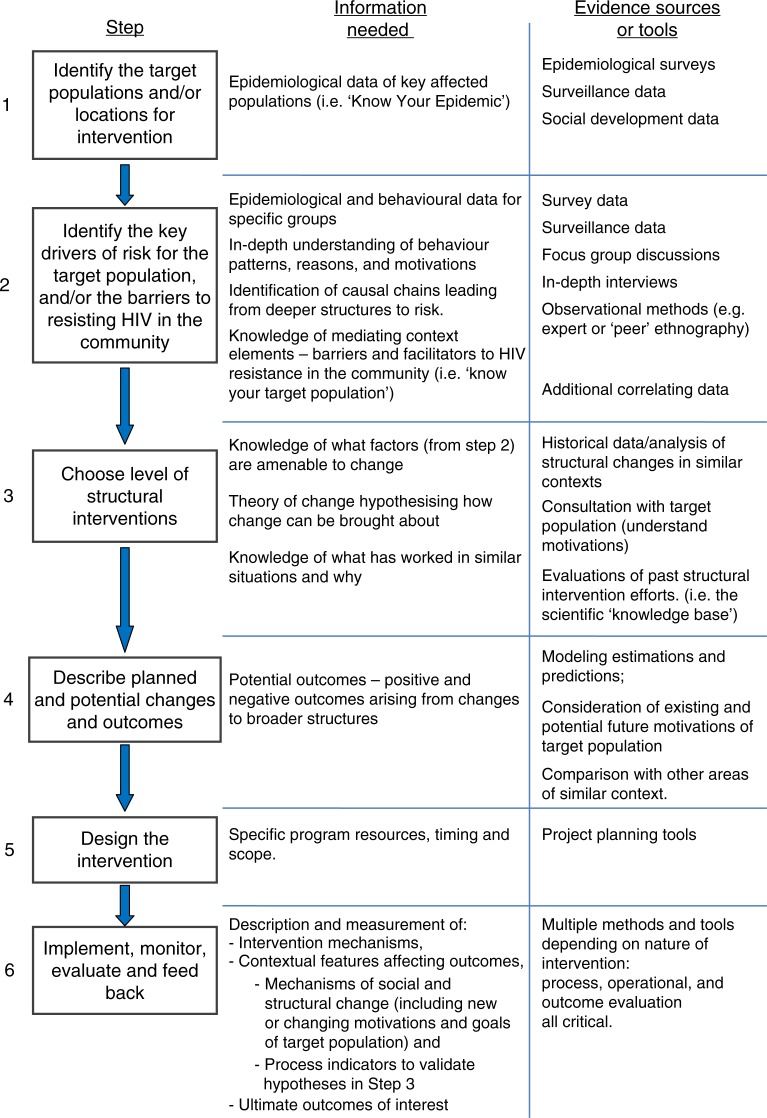
Six-step model: steps and evidence needed to operationalize a structural approach. Adapted from Auerbach *et al*. (2009) [61].

In the preceding section, a structural approach was defined as a process. Operationalizing a structural approach therefore requires following a series of steps and stages, rather than “scaling up” single activities. This is not to say that no interventions from other areas can be useful. The approach proposed here does not say that all HIV prevention interventions must be created from scratch. Instead, interventions must be based on the best evidence of 1) the target population and its risk dynamics and 2) what is known to work for similar risk dynamics elsewhere. Auerbach *et al*. [61] have already presented a six-step approach which was developed in considering structural drivers and causal pathways to help inform a process of decision making in structural responses. This can be adapted slightly to include environmental facilitators and barriers as well as risk drivers, and to further emphasize the ways that intervention planning needs to understand and incorporate the motivations of target populations, as shown in Figure 2.

The six-step model does not predefine interventions (the interventions are not chosen until step 4), but it does select interventions based on what is known from elsewhere, and explicit hypothesizing about its applicability to the local context.

There are, however, a number of specific additional considerations that organizations undertaking structural HIV prevention efforts must bear in mind, including possible unintended consequences, the role of social values within structural planning, consideration of the scope of the programme and issues of generalizability.

***Box 1*. Key definitions of structural HIV concepts from an operational perspective**

- Structural factors – the components other than individual knowledge or awareness which influence individual and group risk and vulnerability.
  - Structural risk drivers – a population-specific subset of structural factors empirically identified to influence individual and/or group risk practices.
    - Causal pathways – the mechanisms through which distal structural drivers lead to proximal influences on the patterning of risk behaviour in particular settings.
  - Structural environmental mediators – a setting- and population-specific set of environmental factors which hinder or facilitate individuals’ and groups’ ability to avoid HIV infection.
    - AIDS resilience – a situation in which individuals possess the capabilities to resist HIV in their given behavioural and risk setting.
- Levels of influence – an operational concept to guide implementing agencies to consider what areas are within their ability to influence. One can look for structural factors influencing the following:
  - Micro – the individual or household level
  - Meso – the community or group level
  - Macro – the broader environment or regional/national level
- Structural interventions – the activities used to address structural drivers in a given setting.
  - For structural risk drivers – those activities which target the structural drivers and their causal pathways for a particular target group.
  - For structural environmental mediators – those activities which build resilience by addressing the environmental factors known to facilitate or hinder individual's ability to resist HIV in their particular context.
- Structural approach – the process undertaken to decide upon an appropriate set of structural interventions.

### Unforeseen or undesired consequences

If attempting to change upstream, distal, structures, it is critical to consider how different patterns of behaviour may arise which can lead to unexpected outcomes. Even with the most thorough consultative process about the current motivations of target populations, an HIV programme official may be unable to predict the future reasoning of the target population given a new structural environment. As such, it is critical for an implementing agency to continually reflect on how individuals and communities are redefining their norms and practices, and the implications this has for HIV transmission. In Figure 1, a hypothesized risk pathway was presented where poverty could lead to isolation from economic opportunities, which may lead to some women engaging in transactional sex. But poverty reduction may open up new HIV risks, as seen in cases where HIV rates are associated with mobility, and as seen in areas where higher HIV prevalence rates have been recorded among wealthier individuals [62].

As causal pathways are varied and can shift, a structural approach should not just hypothesize in advance about what might happen (in step 4), but must further monitor the changing risk environment for the target group to mitigate any new risk situations (within step 6).

### Social change and social values

A related concern for approaches targeting upstream, distal factors involves the implications that shifts in things like gender roles, economic opportunities or laws and regulations will have for other social and political agendas. Poverty reduction may be a common social goal, but the same cannot be said for all changes in economic activities, gender roles or drug laws – all of which can be deeply politicized. HIV prevention agencies may not wish to become political agents, but they need to recognize the implications of structural HIV activities for broader social issues [63].

### Does a single organization need to do everything?

Since a structural approach considers multiple determinants of sexual behaviour, a natural question arises when an organization cannot develop a large-enough body of activities to significantly influence HIV incidence on its own. Even if HIV incidence in a target community is empirically shown to be correlated with a structural factor, there is a risk that HIV prevention actors misleadingly assume that the necessary and sufficient factors shaping social behaviour are those elements the intervention alters (also risking underestimation of the self-directing and adaptable nature of much social practice [46]). In a similar vein, authors who have critiqued the division of HIV prevention programmes into silos of “behavioural,” “biological” or “structural” argue that this risks taking attention away from designing holistic programmes that are more than the sum on individual intervention parts [10, 64, 65]. As such, from the perspective of an HIV planning officer, it may be that the activities conducted are instead seen to contribute to a broader state of AIDS resilience, to provide one of several pieces needed to reduce vulnerability, or to be an essential part of a broader HIV prevention programme of work reliant on equal engagement of target communities. Many may ask if individual activities “work” when they cannot easily be shown to directly reduce HIV incidence on their own. It has been noted that this pressure to show success is one reason that structural approaches to HIV may be neglected [60]. However, as long as there is an explicit and testable hypothesis stated for these interventions, they can be evaluated as to whether they are achieving their interim goals (changing opportunities, improving community resilience, reducing barriers, etc). The ultimate hypothesis about how these structural changes will manifest in changing practices that reduce risk within a target population can then be evaluated over time or in combination with other organizations’ work.

### Generalization and lesson learning

In the social sciences, theories are typically developed to help generalize. When similar outcomes are seen from interventions, and those outcomes can be explained by a plausible mechanism of effect, this is the basis for development of causal theory. A single positive experimental trial result does not establish generalizability, but trials along with other evidence of mechanisms together build the body of evidence from which to work [15, 66–68]. This is why process (or mechanistic) evaluation is so essential in behaviour change interventions and is included in step 6, as shown in Figure 2.

The term sociological plausibility has specifically been used to capture this concept [41]. On the one hand, it is essential to understand local context and to hypothesize why a particular intervention will work for a given population. A particular challenge is to fully comprehend the mechanism of effect, given the active role of target populations reflecting on, considering, and choosing social practices in the face of a changing structural environment. The mechanisms of effect for any given structural intervention (be it access to microcredit, restricted alcohol availability, legal change, etc.) will not be universal, but will be determined by the local context and by the choices target populations make in response to these changes as they decide how to live their lives and achieve their goals[46]. This is why it is particularly important to engage with target populations in the intervention process during tailoring of strategies, to be aware of their goals and reasons for their actions so as to incorporate such insights into intervention strategies, and to learn from them in the evaluations.

At the same time, there will be continued desire to theorize what changes are likely to produce similar results across contexts. Critically, such theorizing must be based on both measures of outcomes and evaluations of causal mechanisms. Although there is still much to be developed in the concept of sociological plausibility – including considerations of when something is plausible enough to expect similar outcomes (when social responses to structural environments are common enough), at the least the concept points to the need to consider why or how a mechanism of effect in one setting might be expected to be similar elsewhere [69].

Based on the above discussions of the nature of structural approaches, and what other factors are important to consider during operationalization, a set of guidelines can be produced on what a structural approach to HIV must, should, and must not do in practice (Box 2).

***Box 2*. Key considerations for a structural approach**

A structural diagnostic HIV approach …

*Must*:

1. Establish which structural factors are influencing HIV risk for the intended beneficiaries
2. Hypothesize the causal chain between intervention and outcome
3. Be aware of possible unintended side effects of upstream changes

*Should* if at all possible:

1. Evaluate key outcomes of the intervention
2. Evaluate the processes by which the interventions did or did not lead to the outcomes seen
3. Monitor how causal pathways may be changing and if new HIV risks or vulnerability may be arising

*Must not*:

1. Alter upstream, ‘structural’ factors without consideration of how they function in the target community
2. Assume a ‘structural intervention’ that showed impact elsewhere will have a similar impact (without considering local similarity or commonality of mechanism)

## Conclusions

For three decades, the HIV prevention community has struggled to reduce the spread of HIV through sexual risk behaviours. This is not to say no successes have been seen. Falling HIV incidence and prevalence in Uganda, in Thailand and in the gay communities in a number of high-income countries, seen in the 1990s, illustrate that prevention can and has worked. UNAIDS has furthermore reported falling global HIV incidence, with 20% fewer new infections in 2011 than in 2001, with the largest declines in the Caribbean and sub-Saharan African regions [70]. Yet where the HIV prevention community has particularly struggled has been in identifying intervention strategies which can replicate such successes.

Some authors have argued for a shift away from this search for interventions to test and, if successful in one setting, scale up. It is explained that an “intervention-oriented” approach proves too limited in its focus on behavioural, biomedical, or, indeed, even structural factors alone. Instead, it is argued to reconceptualize the unit of analysis to be that of HIV prevention programmes (which will undoubtedly need to integrate multiple interventions and continually adapt), rather than single interventions [64]. Here, this paper retains structural approaches as a unit of analysis, but it shares the conceptual concern of this “Program Science” approach [65], which recognizes the limitations of single interventions, and refocuses efforts on the ultimate objective of HIV prevention efforts – population wide incidence reduction [65, 71].

Biomedical sciences have shown a number of recent breakthroughs in the field of prevention. But the science of behaviour change is a social science, and to move forward, the HIV prevention community must learn how to incorporate the social science lessons about behaviour which have been known for decades, but which have yet to change HIV planning. Future HIV prevention efforts must address the multiple structural factors shaping risk and vulnerability, and they must do so in ways tailored to particular settings.

Epidemiological studies have shown that, time and again, single, predefined behaviour change interventions, delivered in short time periods, typically are unable to achieve these things [40]. So far, the answer to this disconnect has effectively been “keep looking” – a re-emphasis on the desire to find single, predefined interventions which can work, in the face of the theory and evidence that these types of interventions do not align with how human behaviour functions. The field of HIV prevention is changing, however. There is greater understanding of the limitations of past approaches, greater acceptance of complexity and more calls for combinations of strategies. This period of change provides a window of opportunity to define and establish best practices for structural approaches to ensure that they address the key social insights about HIV risk and vulnerability.

Programme implementers must consider a number of questions to guide their activities. Such questions may include:

1. What target group(s) is the intervention trying to influence?
2. At what level does your agency work?
3. What is the range of potential ways your group can act?
4. What time point are you working towards?
5. What is your theory of change, and what can you feasibly contribute to achieve change? This should also include:
  - What factors are outside your area of control, and how are you expecting communities to react to any changes you engender?
6. What can you measure and monitor as part of your activities?
7. How important is it to show impact on HIV incidence (versus contributing a component to a larger response)?

There are already examples of structural interventions which appear to be based on a diagnosis of what is driving HIV risk in a target population group. The Avahan project, which addressed the risk environment for sex workers in Kolkata, is often cited as a programme designed to respond to local needs, rather than imposing top-down interventions [44] (with attempts being made to try to emulate its success in scaled-up settings [72, 73]). In South Africa, recognition of the importance of alcohol use in influencing risky sex led to an HIV and alcohol linked-skills programme which achieved a 65% reduction in unprotected sex (compared to a control group receiving HIV education alone) [74]. Similarly, a number of cash-transfer programmes have arisen in settings where young women are known to engage in transactional intergenerational sexual relationships [75–77]. These programmes may not have followed all the steps recommended in this paper, but they do provide an indication that targeting structural factors in a tailored way is indeed feasible. What has been lacking, however, is a systematic or widely agreed-upon HIV prevention approach that ensures appropriate contextual leaning and tailoring of interventions.

Hunsmann's work illustrates the institutional incompatibility of many organizations to taking up structural HIV prevention strategies [60]. He notes that the political attractiveness of policies depends on the nature and speed of results they can achieve, that the fragmented and vertical nature of development assistance is not conducive to structural approaches and that policy makers also perceive structural approaches as too complex. The author further notes the institutional path dependency of many agencies makes changing strategy particularly hard.

There are further challenges as well to achieving a shift in the status quo of HIV prevention. The introduction noted that it is intuitive for many stakeholders to look at successful cases of HIV prevention and attempt to copy the activities conducted in other settings. Such intuition no doubt arises from human cognitive reliance on simplifying heuristics and predispositions to look for similarities or create causal explanations [78, 79]. Further, the majority of individuals working in public health today have been trained in disciplines grounded in positivist approaches, such as clinical medicine, in which the objects of study (e.g., pathogens) are not conscious reflective agents (as people are), and in which (biochemical and anatomical) similarities across populations are often taken for granted resulting in a typically unquestioned assumption of external validity when an intervention is shown to produce a causal effect. Shifting this mindset is therefore doubly challenging – requiring both a conscious awareness of our own conceptual biases, as well as an epistemological paradigm shift to recognize the inappropriateness of clinical reasoning and knowledge for understanding fundamentally social phenomenon like human sexual behaviour.

Although public health actors appear to have struggled to take on the social science learning of the past, the institutional norms of programme managers may now provide a better target for the HIV prevention community. Public health institutions, while facing the difficult incentives described above, do also typically look to identify best practices to use as standards. As such there is a need for the HIV prevention community to collectively define “good practice” for HIV prevention in a way that ensures interventions follow a locally relevant process, rather than a decontextualized selection of activities. Future HIV prevention work that fails to have an explicit and well justified theory of change, or which continues to rely on education messaging alone (not part of a broader programme of work), should equally be branded “bad practice” for HIV prevention. Just as clinical authorities denounce programmes utilizing substandard treatments, or epidemiologists reject poorly designed trials as invalid and unethical, good practice in HIV prevention requires new standards to which programme officers can refer, rather than additional knowledge we hope they will incorporate.

Institutional change is not something that a donor-funded working paper, a journal special issue or well-reasoned argument can bring about on its own. Instead, institutions change when new rules, norms or binding expectations are established. Existing funding sources and public expectations may currently provide institutional pressure to continue HIV programming as usual – leading to short term, oversimplified, information-driven prevention strategies. This pressure can only be countered by establishing globally accepted best practice guidelines which point out how those approaches are insufficient, while providing clarity on alternatives for the future. It is hoped that this paper can provide an important step to contribute to an ongoing discussion through which such global best practices to improve HIV prevention efforts can be developed.
